# Supplementary material for: Transcriptome and Metabolite Profiling of Tomato SGR-Knockout Null Lines Using the CRISPR/Cas9 System
Source: Int J Mol Sci. 2022 Dec 21;24(1):109. doi: 10.3390/ijms24010109 (PMC9820150; doi:10.3390/ijms24010109)
Supplement: Supplementary file 1 [file ijms-24-00109-s001.zip › ijms-2038112-supplementary.pdf]

Supplementary Data

Transcriptome and Metabolite Profiling of Tomato SGR-Knockout Null Lines Using the CRISPR/Cas9 System

Jin Young Kim, Jong Hee Kim, Young Hee Jang, Jihyeon Yu, Sangsu Bae, Me-Sun Kim, Yong-Gu Cho, Yu Jin Jung and Kwon Kyoo Kang

Contents

Table S1. Design of sgRNAs for CRISPR genome editing on *SlSGR1* gene in tomato using the CRISPR RGEN tool program (http://www.rgenome.net/).

Table S2. Detection of mutations on the putative off-target sites in edited plants.

Table S3. Oligonucleotide primers for mutation analysis in the putative off-target sites in edited plants.

Table S4. Determination of carotenoid contents in *sgr1* #1-6, *sgr1* #2-4 and WT leaves.

Table S5. Primer list, oligonucleotide sequence and purpose used in this study.

Table S6. Log2 fold change of metabolite between the *sgr1* #1-6 and wild type control.

Figure S1. Resequencing analysis of gene edited plants. a Mapping of genome sequences of transgenic plant contained b mapping of genome sequences of *sgr1* #1-6 lines, c mapping of genome sequences of *sgr1* #2-4 lines, Light blue highlights indicate the annotated vector regions. The solid red line marks the average genome coverage, and the dashed lines are the half and the double of the coverage. The T-DNA, which is enclosed by left and right borders (LB/RB), shows 20× coverage as expected for a single copy locus.

Supplementary Table S1. Design of sgRNAs for CRISPR genome editing on *SlSGR1* gene in tomato using the CRISPR RGEN tool program (http://www.rgenome.net/).

SGR1	sgRNA Target (5' to 3')	Direction	GC Contents (%, w/o PAM)	Out-of-frame Score	Mismatches			
					0	1	2	3
sgRNA1	GTCCATTGCCACATTAGTGGAGG	+	50.0	62.9	1	0	0	0
sgRNA2	CCCCAGTGAGTGTTATGCCTTGG	+	55.0	70.9	1	0	0	0

Supplementary Table S2 Detection of mutations on the putative off-target sites in edited plants.

Target	Putative off-target	Off-target locus	Sequence of off-target site	No. of mutation plants
sgRNA 1	OFF 1	ch01:24349961-24349683	GaCCcTTGCCAtATTcGTGGAGG	0
	OFF 2	ch03:14040183-14040205	GTCCATTGaCtgATaAGTGGAGG	0
	OFF 3	ch04:38450518-38450540	GaCCATTGCCACAccAcTGGAGG	0
	OFF 4	ch08:1675678-1675700	GTCCAcaGCCACgTTAcTGGGGG	0
sgRNA 2	OFF 1	ch07:17871427-17871449	CgttAGTGAGTGcTATGCCTAGG	0
	OFF 2	ch07:35441694-35441716	CattgGTGAGTGTTATGCCTAGG	0
	OFF 3	ch10:54619271-54619293	gCCCAtTGAGTGTTcTaCCTCGG	0

Supplementary Table S3. Oligonucleotide primers for mutation analysis in the putative off-target sites in edited plants.

Off-target site	Primers	Sequence (5'→3')
sgRNA 1	FW	AAATTCAGAATGCCCTGTCG
OFF1	RV	TCATGAACAGTTCCCGATCA
sgRNA 1	FW	AACTGTAAAAGCGCCCATTG
OFF2	RV	GGAATCAGTGTGTGGTGTCG
sgRNA 1	FW	TGGCTCAAGCTCAATCAGAA
OFF3	RV	AGGCGATCCATAAGACGATG
sgRNA 2	FW	ATCATACACTGCCGCTTGTG
OFF1	RV	TAAGGCTTGCTGGTTTTTGG
sgRNA 2	FW	TGTGGGGGTATCTAGCCAAG
OFF2	RV	TGTTTCCTGGAGGAAGATCG
sgRNA 2	FW	TGTAGGCGCTTTCCTGAAGT
OFF3	RV	CATCCCCGATTAACAGAACG

Supplementary Table S4. Determination of carotenoid contents in *sgr1* #1-6, *sgr1* #2-4 and WT leaves.

samples	violaxantin		lutein		zeaxantin		13*Z*-*β*-carotene		*α*-carotene		*β*-carotene		9*Z*-*β*-carotene		others		total	
	mean	SD	mean	SD	mean	SD	mean	SD	mean	SD	mean	SD	mean	SD	mean	SD	mean	SD
WT	221.3	11.9	925.2	39.3	22.6	2.4	37.0	1.3	35.0	0.5	607.7	24.3	92.5	6.6	543.4	22.2	2484.6	108.5
sgr1 #1-6	527.1	12.4	311.7	20.4	74.5	5.3	41.1	2.7	36.1	2.9	844.6	6.0	121.3	5.1	379.5	11.3	2335.9	266.2
sgr1 #2-4	589.4	3.7	352.8	94.1	68.2	7.4	47.7	3.9	39.5	4.4	944.2	98.5	135.1	19.8	267.4	53.5	2444..3	285.3

*Data are expressed as mean (the average value of content for dry weight) and SD (the standard deviation value) of three independent experiments. Carotenoid contents were calculated as μg g-1 dry weight of leaf tissues.

Supplementary Table S5. Primer list, oligonucleotide sequence and purpose used in this study.

Primers	Sequence (5'→3')	Purpose
sgRNA1 up	gattGTCCATTGCCACATTAGTGG	Vector construction
sgRNA1 down	aaacCCACTAATGTGGCAATGGAC	
sgRNA2 up	gattCCCCAGTGAGTGTTATGCCT	
sgRNA2 down	aaacAGGCATAACACTCACTGGGG	
pBOsC sgSEQ Fw	CAGCTTGGCTCTAGTCGACC	Confirm of sgRNA in pKAtC vector
sgRNA scaffold region	cggtgccactttttcaagtt	
Kanamycin-R Fw	ATGATTGAACAAGATGGATTGCAC	Transgenic plant identification
Kanamycin-R Rv	TCAGAAGAACTCGTCAAGAAGGC	
NGS sg1 1st Fw	AAATCCCACACATCACATGC	NGS analysis
NGS sg1 1st Rv	TCAAGGCTTTTGTTCATGGA	
NGS sg1 2nd Fw	acactctttccctacacgacgctcttccgatctTTGCAGTTGCAAGGTTGGTA	
NGS sg1 2nd Rv	gtgactggagttcagacgtgtgctcttccgatctATGTCGTTCGAACTTCCCAA	
NGS sg2 1st Fw	GGAATTATCCAGAGTTACAAGAAGC	
NGS sg2 1st Rv	GTTGGGTTGTGCCTAAATCAA	
NGS sg2 2nd Fw	acactctttccctacacgacgctcttccgatctGGGTAGGTGGGGTGAAGAGT	
NGS sg2 2nd Rv	gtgactggagttcagacgtgtgctcttccgatctTGTTTGGTTTATTATGTGATTTGATG	
SlSGR1 Fw	TTCTTCTGGTGGGGTAGGTG	

SlSGR1 Rv	AGGCATAACACTCACTGGGG	
SlPSY1 Fw	TAGCACAGGCAGGTCTATCC	
SlPSY1 Rv	CCTTTCTCTGCCTCATCAAA	
SlPSY2 Fw	TAGCACAGGCAGGTCTATCC	
SlPSY2 Rv	GTCGTTTGCTTCAATCTCGT	
SlPDS Fw	ATTTAGCCGGTGACTACACG	
SlPDS Rv	CCGACAACTTCTTTTGGCTA	
SlGGPS Fw	AAAATACGGTCGAGCTGTTG	
SlGGPS Rv	AATGCCATAAACGCTGACAT	
SlZDS Fw	GGACCTGGTAAAGACCCATT	
SlZDS Rv	TGCCTACCTGAAAGAGTTGC	
SlCRTISO Fw	GACCCACAGACGATACCTTG	RT-PCR analysis
SlCRTISO Rv	CTTGTCCTGGGAAGCAACTA	
SlLCY B Fw	TGCTGGTATGGTTCATCCTT	
SlLCY B Rv	CCAAACAGATGCCGATAACT	
SlLCY E Fw	CTACGATTGAACACCCTTGG	
SlLCY E Rv	ATAACCTGTGGCTGGATGAA	
SlPSY3 Fw	TAGACCTCGAGAATCGTTCG	
SlPSY3 Rv	CCACAATTACGCCTCAGTTC	
SlFabG Fw	CCGTTGAGAACACATGGAAC	
SlFabG Rv	GATGAGAAAATCGGGACACC	
SlAP2a Fw	TGAAGTTGAAGCTGCCAGAG	
SlAP2a Rv	TGGTCCGTTGCATTATCAGT	
SlDDTFR8 Fw	CTTGACGCATTCCAGTGTTT	

SlDDTFR8 Rv	GCCCTTTCAGATCCTTCAAA
SlRIN Fw	ACATCATGGCATTGTGGTG
SlRIN Rv	GCTGCATTTTCGGGTTGTA
SlLOXB Fw	GGTGGCCTAAACTGCAAACT
SlLOXB Rv	GGCGATTAGGGAGATAACCA
SlERF-D2 Fw	CTGCATTGAGATTTCGAGGA
SlERF-D2 Rv	TTAGAGGGGATGGATTGGAG
SlloxC Fw	GGGTAACTTCTGGCCATCAT
SlloxC Rv	CAAGAACCACTCCCATTCCT
SlACO1 Fw	GCGCCACTCTATTGTGGTTA
SlACO1 Rv	TGCATCACTTCCTGGATTGT
SlACO6 Fw	GCCACTCCATTGTCATCAAC
SlACO6 Rv	AACGAAGCCAAGGACATTCT
SlACTIN – FW	GGGATGGAGAAGTTTGGTGGTGG
SlACTIN – RV	CTTCGACCAAGGGATGGTGTAGC

Supplementary Table S6. Log2 fold change of metabolite between the *sgr1* #1-6 and wild type control.

Metabolite	*sgr1* #1-6 / WT leaf	*sgr1* #1-6 / WT Fruit
Sucrose	0.24	-2.13
galactinol	2.11	1.86
Raffinose	0.42	0.29
Glucose	0.15	0.62
Xylose	1.12	0.73
Fructose	0.52	0.23
Dehydroascorbate	0.21	0.98
Galacturonate	0.24	0.87
Rahamnose	0.91	1.25
G6P	-1.74	1.64
erythritol	0.08	-1.58
myo-inositol	1.11	-1.54
saccharate	0.01	-2.24
glucar.lac	-0.54	0.89
Trehalose	1.46	-0.48
Maltose	0.41	-0.49
maltostriose	-1.55	2.05
F6P	0.91	-1.35
tryptiophan	0.67	-1.29
Phenylalanine	0.48	-0.37
caffeoulquinic acid	-2.31	2.18
caffeate	-0.31	-1.17
Valine	0.58	0.61
Alanine	0.37	1.21
Leucine	2.04	1.51
Citrate	2.15	2.07

Glutamate	-0.13	0.48
Ornithine	1.87	1.91
Putrescine	2.08	0.01
4-Aminobutyrate	0.34	1.88
Glutathion	2.31	1.77
Fumarate	1.24	0.46
Malate	-0.03	-0.58
Aspartate	0.61	1.14
Asparagine	2.11	1.79
β-alanine	2.06	1.99

*Student's *t*-test was performed to compare *sgr1* #1-6 with wild type ($P < 0.05$; n = 5)

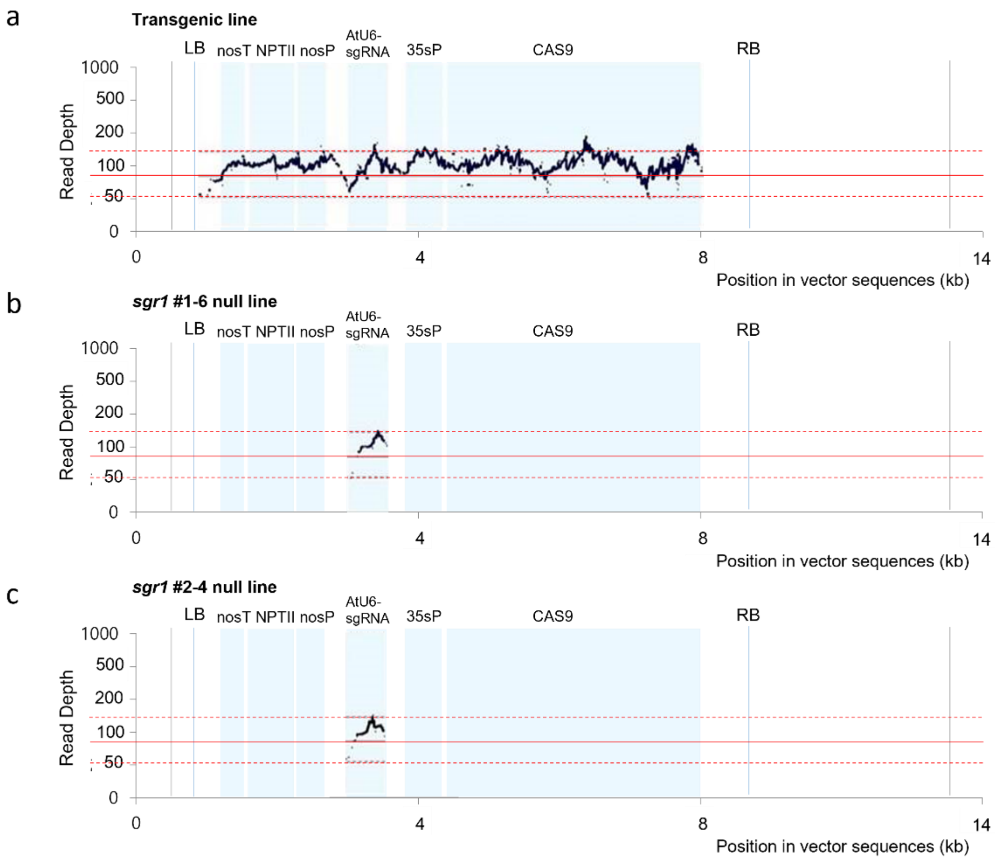

Supplementary Figure S1. Resequencing analysis of gene edited plants. a Mapping of genome sequences of transgenic plant contained b mapping of genome sequences of *sgr1* #1-6 lines, c mapping of genome sequences of *sgr1* #2-4 lines, Light blue highlights indicate the annotated vector regions. The solid red line marks the average genome coverage, and the dashed lines are the half and the double of the coverage. The T-DNA, which is enclosed by left and right borders (LB/RB), shows 20× coverage as expected for a single copy locus.
